# De-noising of dynamic magnetic resonance images by the combined application of wavelet filtering and Karhunen-Loeve Transform (KLT)

**DOI:** 10.1186/1532-429X-14-S1-W71

**Published:** 2012-02-01

**Authors:** Prashanth Palaniappan, Orlando P Simonetti, Yu Ding

**Affiliations:** 1 Department of Electrical and Computer Engineering, The Ohio State University, Columbus, OH, USA; 2 The Ohio State University, Columbus, OH, USA

## Background

Real-time dynamic magnetic resonance imaging (MRI) typically sacrifices signal-to-noise ratio (SNR) to achieve higher spatial and temporal resolution. Spatial and/or temporal filtering methods can be used increase SNR, but at the expense of edge sharpness, i.e., image blurring.

## Objective

In our current study, we describe a new approach to filtering dynamic image series that aims to remove noise without blurring stationary/ moving edges and requires no training data.

## Methods

The proposed approach combines 2D spatial wavelet filtering with 1D temporal Karhunen-Loeve Transform (KLT). The KLT is first applied to create a series of “eigenimages” in which important signal information is concentrated into only a few eigenimages. Then a 2D spatial wavelet filter is applied to each of the individual eigenimages. An adaptive threshold is used to define the wavelet filter strength for each of the eigenimages based on the noise variance and standard deviation of the signal, resulting in stronger filtering of the eigenimages that primarily contain noise. After wavelet filtering, the de-noised eigenimages are transformed back into image space.

The performance of this filtering approach was tested over a range of wavelet filter strengths (1x to 4x adaptive threshold) for noise reduction and edge sharpness in a digital phantom. SNR improvement was also evaluated in real-time stress cine image series acquired in five normal volunteers. SNR was calculated in phantom and human images by finding the mean signal value and noise variance in a region-of-interest (ROI) within each frame. The sharpness in phantom images was measured as the distance between 20% and 80% of the total rise/fall for a moving edge.

## Results

For the digital phantom images, it is evident from the SNR plot that the proposed algorithm gives better results compared to 3 other filtering techniques- spatial wavelet filtering alone, temporal KLT filtering and spatial Gaussian 2D low pass filtering.

From sharpness measurements done on the phantom images, it was observed that the proposed algorithm preserves stationery edge sharpness better compared to spatial filters (wavelet and Gaussian) and preserves moving edge sharpness better compared to temporal filters (KLT). For real-time stress cine images, the SNR improvement was 61.75% for wavelet threshold*1, 78.07% for threshold*2 and 94.65% for threshold*3.

## Conclusions

Noise reduction in real-time cine images of up to 62% +/- 17% is feasible without visible edge blurring using the proposed method of spatial wavelet filtering in the temporal eigenimage domain. Future studies will investigate the application of this method in other SNR-challenged dynamic acquisitions such as first-pass perfusion and real-time phase contrast.

## Funding

R01HL102450.

**Figure 1:**
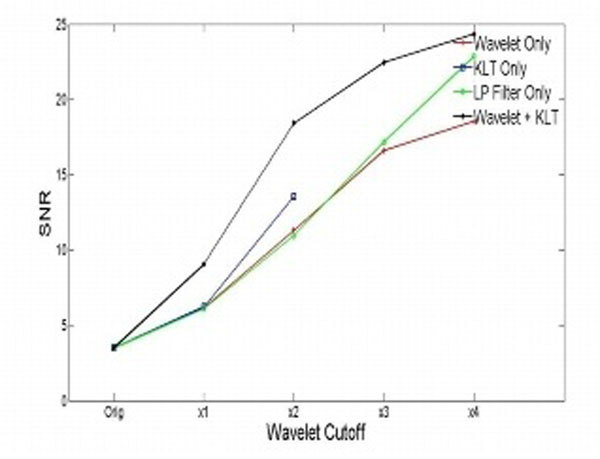
SNR characteristics of different filtering techiques for digital phantom data

**Figure 2:**
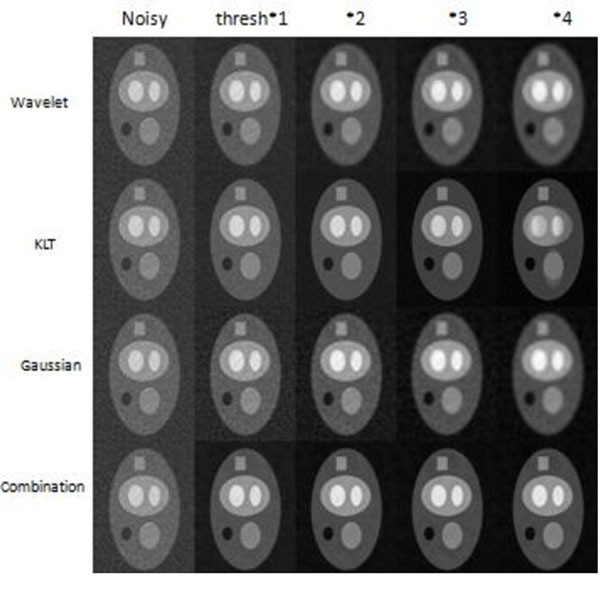
Performance of different filtering techniques

